# How to Help Your Complex Patient If You Don't Know What Is Wrong

**DOI:** 10.1177/23743735221133639

**Published:** 2022-10-30

**Authors:** Sarah Clark

**Affiliations:** 1 Geography, University of Southampton, Southampton, UK; 2 Clinical and Developmental Neuropsychology, 6657 Bournemouth University, Poole, UK

**Keywords:** therapeutic clinical encounter, doctor–patient relationship, clinical encounter, healthcare interactions, perceptions, continuum of care, person-centeredness, partnership

## Abstract

Perspectives from a complex patient point-of-view on how clinicians can improve therapeutic empathy skills and how to help a complex patient who is undiagnosed and you do not know what is wrong.

What if you are a doctor with a very complicated patient troubled by undiagnosed, disabling, and debilitating symptoms but you just do not know, as their doctor, what could be wrong or how to treat it? What do you do then? How do you not let your years of medical training confound your clinical judgment when it comes to applying this knowledge to your real-life patient in front of you? And how can you avoid medical gaslighting?

As a complex patient, I have strong opinions about what a “*good doctor*” is and I can only imagine how I must scare or confuse many of my doctors. I am studying part-time for a Master’s degree in Clinical and Developmental Neuropsychology and I know my academic studies have helped me become a much better patient in how I communicate with my doctors. I aspire to a future career in medical research, and I am increasingly managing to bridge the academic skills from the taught course content with my own lived experience as a complex patient. I have also undertaken voluntary work in patient advocacy and medical education.

Through my own diagnostic journey, I’ve become interested in misdiagnosis, the difference a diagnosis makes, and trauma from the medical system as well as the language and communication skills used in healthcare settings.

It took some 15 years from my original physical symptom onset until I was diagnosed in February 2020 with hypermobile Ehlers–Danlos syndrome, and subsequently Dysautonomia, Median arcuate ligament syndrome, and Mast cell activation syndrome. All this is upon a plethora of past psychiatric misdiagnoses (including a personality disorder) which confounded my diagnostic journey with my physical health issues. I also have Complex Post-traumatic stress disorder (C-PTSD) and Obsessive compulsive disorder (OCD). I was additionally diagnosed with Autism spectrum disorder (ASD) and Premenstrual dysphoric disorder (PMDD) this year.

From years of clinicial encounters (both good and bad) as a complex patient, I have come to rate my doctors and categorize them as follows:

1. Doctors with poor clinical knowledge and poor communication skills who subject their complex patients to gaslighting, misdiagnosis, or unnecessary treatment. As a patient you are unable to get your voice heard above the expert. Doctors I’ve encountered in this category have blamed what I knew were physical symptoms on my emotional problems and I was misbelieved. One personal example I can use here to illustrate was when I attended A&E having fallen off a ladder and I was tearful due to the pain. I was not examined physically, simply told to go home and phone the mental health crisis team because the doctor said I looked distressed. He totally disregarded the fact I had had a genuine accident. I then lived with severe lower back pain for the next four years, not even able to sit down properly. I only discovered my spine was dislocated in 2 places when in desperation I had a private upright MRI scan four years after the accident. The spinal dislocation was then corrected by a very experienced hypermobility physio.
2. Doctors with lesser clinical knowledge, but compensated for with excellent communication and empathy skills- kindness and compassion really can make a difference when you are suffering.
3. Those with outstanding clinical knowledge (often the academic clinians) and excellent communication skills and those able to take a much more holistic view of the whole person.

I often find it is the doctors who are the mavericks, non-conformists, and revolutionists, who can *think outside the box*, be adaptable in how they put their education and training into clinical practice, apply their textbook knowledge to the uniqueness of each real-life patient, and those who are open-minded and willing to learn who make the *best doctors*.

Conversely, in my experience it's those with poor communication skills, poor knowledge and unwilling to admit they “don't know” and unwilling to learn anything new that blame the patient for their clinical knowledge insufficiencies, say there is nothing wrong or think they patient is neurotic rather than be prepared to see any deficiency in knowledge within their own clinical skillset.

Effective healthcare seems to be most successful if the patient is seen as unique with a unique spectrum of multi-dimensional needs including medical, mental health, and socio-economic (1).

Making a diagnosis can be a fine balancing-act between the consideration of formulating a medical explanation for someone's struggles and suffering within their unique human experience, versus the potential risks medicalization could bring. This may include transforming people into patients and potentially turning patients into consumers, thus de-humanizing the human experience. Jones et al (2) describe how further post-diagnostic issues also can include discrimination, inadequate support or treatment availability, unnecessary medication, and iatrogenic harm (harm caused inadvertently by a doctor or by medical treatment or diagnostic procedures).

To many clinicians, a diagnosis is often purely about being able to provide treatment or medical interventions. However, the transformative difference a diagnosis can make to a patient can often involve learning about their condition, learning how to self-manage, and creating a new self-identity and self-narrative. These are things doctors seem to poorly understand.

From my patient advocacy training and work, I strive that my own *healthcare* should be *teamwork* including treating me as a *person* and humanizing my healthcare, not just treating my presenting symptoms, and not just a thing that is done *to* the patient. This teamwork needs to involve open, respectful, and sensitive communication to facilitate a meaningful and therapeutic doctor–patient relationship with the primacy of healing relationships in care provision and to strive towards a more holistic patient-centered approach (3).

What I mainly hope to highlight here is the top-down hierarchical provision from the majority of doctors, undermining patients who are the ones with the lived-experience of the condition. Often it seems it is the most complex patients who carry the burden of being left to figure things out for themselves in their diagnostic odyssey if their clinicians lack sub-specialist knowledge (4).

All too often I have been at the receiving end of the dichotomous power construct of the modern healthcare system where I have been medically gaslighted, unable to get my voice heard above the expert opinion of my doctors, and subjected to structural iatrogenic harm from years of medical invalidation where many of my doctors tried to simply negate my symptoms, blaming them on being linked to my past traumas and psychological issues. This level of medical invalidation only served to intensify the psycho-social difficulties I already faced.

I have often been repeatedly faced with my doctors saying that there “*is no point in carrying out tests for these symptoms because it would not make any difference to the managemen*t.” However, in patients desperately seeking care within the biomedical model, a diagnostic apotheosis with a clinical diagnosis is of fundamental relevance when considered within the context of the psychology of chronic illness identity and the identification of a medically valid explanation for suffering.

From my experience, how “to be a good doctor” also stems not only from their clinical acumen, but also from the clinician's personal characteristics. How empathetic they are as a person, their listening skills, compassion, interpersonal understanding, and caring actions, and how these can be amalgamated within their *therapeutic empathy* (5).

Repeated *unsuccessful* clinical encounters often create stress, tension and antagonism (4). Whereas *therapeutic empathy*, seen as the tallying between empathy and person-centered care (6) goes a very long way to a meaningful and successful clinical encounter.

The structural doctor–patient biases (11) in the healthcare system can fuel antagonism in the patient–provider relationship and dissatisfaction with care provision. Indeed, Stacey et al (7), instead located these challenges in the “interactional encounter” itself as opposed to placing the blame on the *difficult patient*.

Navigating through the ever-changing complex medical lexicon and nosology, weighing up the fear of a *missed* diagnosis versus a *misdiagnosis* is a tricky clinical skill to master in practice, especially when litigation is becoming increasingly rife.

Where a condition remains undiagnosed (perhaps due to sub-clinical symptoms) for many years resulting in dismissal, skepticism and disbelief on the part of the clinician, often doubting the validity and reality of the patient's presentation of their symptom extent. This can lead to repeated and frustrating clinical encounters which delegitimize a patient's experience and identity. It is not uncommon for this frustration to manifest for the more complex of patients in being misdiagnosed with a plethora of harmful labels such as “neurotic depression.” This may even result in patients questioning their own sanity, experiences, perception, and identity. Perhaps even resulting in iatrogenic anxiety, depression, or medically induced Post-traumatic stress disorder leading to hypervigilance and future avoidance of seeking crucial healthcare due to past invalidation.

I find much solace in a doctor who is prepared to admit if they do not know, and refer to a colleague who *might* know.

When working with a patient who has had a long journey through the medical system, and faced much trauma and invalidation, there is often a need on the part of the clinician to learn how to regain a patient's trust, particularly if they have been previously misdiagnosed or felt unheard (8).

While a vast number of factors can determine the perception of successful clinical encounters, considering both the nocebo effect doing more harm than good (9) and the social, physiological, and emotional consequences of invalidating communication when compared to validating communication (10) could be one way to improve successful outcomes in clinical encounters.

Many doctors also need a better appreciation that the medical literature is often on average 17 years behind the research world and not to take such a negative view if your complex patient might know more than you on a particular topic having done some reading on “Dr Google.” We need to view the evidence as a tool, and not just as a determinant of how medicine should be practiced (12). Healthcare is about people, not just illness.

For many patients a *good doctor* is far less just about what medication they can think to prescribe, it is about being heard and listened to by your doctor and viewing a patient as a human being not just a list of symptoms to medicate.

From my perspective as a complex patient, highly traumatized by iatrogenic harm caused by the structural imbalances in the healthcare system, we should strive towards a paradigm shift in the cognitive dissonance whereby doctors fail to wholly comprehend the lived realities and everyday experiences of many of the chronic conditions. In my view, this could in-part be achieved by incorporating more lived-experience sessions in medical education settings. Likewise, clinicians need to gain a deeperunderstanding of the reasons and barriers to self-care and the interaction of managing several comorbidities as well as joining up the dots and seeing a patient as a whole person rather than just an amalgamation of discrete body systems which are to be treated individually.

Therapeutic empathy is something it is possible to develop and cultivate, not an untrainable reflex (5).
